# Identification of Suitable Biomarkers for Stress and Emotion Detection for Future Personal Affective Wearable Sensors

**DOI:** 10.3390/bios10040040

**Published:** 2020-04-16

**Authors:** Abdulaziz Zamkah, Terence Hui, Simon Andrews, Nilanjan Dey, Fuqian Shi, R. Simon Sherratt

**Affiliations:** 1 Biomedical Sciences and Biomedical Engineering, The University of Reading, Reading RG6 6AY, UK; a.a.a.zamkah@pgr.reading.ac.uk (A.Z.); t.k.hui@reading.ac.uk (T.H.); s.c.andrews@reading.ac.uk (S.A.); 2 Department of Information Technology, Techno India College of Technology, West Bengal 700156, India; neelanjan.dey@gmail.com; 3 Rutgers Cancer Institute of New Jersey, Rutgers University, New Brunswick, NJ 08903, USA; fuqian.shi@rutgers.edu

**Keywords:** stress, emotion, cortisol, volatile organic components, biomarkers, wearable sensors

## Abstract

Skin conductivity (i.e., sweat) forms the basis of many physiology-based emotion and stress detection systems. However, such systems typically do not detect the biomarkers present in sweat, and thus do not take advantage of the biological information in the sweat. Likewise, such systems do not detect the volatile organic components (VOC’s) created under stressful conditions. This work presents a review into the current status of human emotional stress biomarkers and proposes the major potential biomarkers for future wearable sensors in affective systems. Emotional stress has been classified as a major contributor in several social problems, related to crime, health, the economy, and indeed quality of life. While blood cortisol tests, electroencephalography and physiological parameter methods are the gold standards for measuring stress; however, they are typically invasive or inconvenient and not suitable for wearable real-time stress monitoring. Alternatively, cortisol in biofluids and VOCs emitted from the skin appear to be practical and useful markers for sensors to detect emotional stress events. This work has identified antistress hormones and cortisol metabolites as the primary stress biomarkers that can be used in future sensors for wearable affective systems.

## 1. Introduction

For many years, scientists have known that emotions can be communicated among animals by changing their body odors [1]. In stressful events, such as being injured or in life-threating situations, chemical biosignals are released from the skin to warn other animals to escape or to gather. For example, Valenta and Rigby [2] showed that rats can differentiate between stressed and relaxed rats using airborne odor. Therefore, it has been postulated that such effects may be extended to humans. Many experiments have been conducted to determine the role of human odors in emotional communication. Consequently, it is now known that humans can smell several emotions, including happiness [3], fear [4], and anger [5]. Indeed, Benderly [6] stated that “olfaction is our most emotional sense”.

In addition to body odor, physiological changes (such as heart rate, skin conductivity, and oxygen saturation) in the human body occur as an emotional response. Hui and Sherratt [7] used physiological sensor data to detect emotional events based on the concept of emotional context awareness. Happy and Routray [8] used image processing to detect emotional states in facial expression. Li et al. [9] merged facial image processing with electroencephalography (EEG) for improved emotional state detection, indicating that affective systems benefit from being multimodal. Yang et al. [10] demonstrated emotion detection through speech for AI-based home assistants. While there is a large research area around affective systems and their impact on emotion and stress, this paper will review the literature, specifically looking for identified biomarkers in sweat that could be used to improve future affective sensors’ sensitivity or classification to detect stress and emotion.

In the modern era, stress has been identified as a significant factor that affects health, the economy, and quality of life [11,12,13,14]. Researchers have recognized the relationship between the emotions of an individual and their health [13], which in turn has raised the subject of recognizing emotional status through affective computing [15]. These emotions were classified by some researchers into six basic emotions, namely fear, disgust, joy, anger, sadness, and surprise [13]. Recently, stress has been added to the recognized emotion set, which can be defined as the feeling caused by emotional tension, which might happen in certain circumstances when one has to react to demand or pressure that does not match with knowledge and experience, or is over their capability [11,16]. In the modern world, stress is a crucial problem. For example, researchers have reported that a growing number of community violence cases are related to anger resulting from stressful experiences [5,17,18]. Furthermore, police officers who do not cope with stress and its consequences have been shown to have increased rates of post-traumatic stress disorder and increased aggression [17]. Also, stress has been shown to harm human health and plays a key role in diseases related to a mental disorder, such as anxiety [19] and seizures [14,20]. Because of these risky influencers of stress, researchers have focused on overcoming the issues and detecting stress as early as possible to prevent further development. Although the classic invasive blood cortisol tests are the gold standard for measuring stress, there are two major methods that have been used to detect stress noninvasively, either measuring brain waves via implementing EEG electrodes or utilizing biomedical tools to detect physiological biosignals, such as heart rate (HR), blood pressure (BP), and body temperature, and by using sweat sensors to measure skin conductivity (SC) [21]. In terms of device wearability, although EEG provides accurate readings and valuable information about the brain’s states, its main disadvantage is that EEG electrodes must be attached to the scalp. which is reported to be inconvenient for users [22]. While SC sensors are common in emotion detection systems, they are mainly used for measuring skin conductivity rather than the electrochemical content of the sweat. Sweat’s electrochemical contents, such as the stress hormone cortisol, and skin gases are significantly under-researched.

This review considers the current state of the art in the understanding of biomarkers present in sweat under stress and emotional events. We present the most recent electrochemical sweat markers and skin VOC studies to hypothesize potential stress biomarkers for future affective technology sensors.

Section 2 presents our research methodology. Stress sweating physiology and stress electrochemical biomarkers are discussed in Section 3. A review of gas emissions from the skin, known as volatile organic components (VOCs), during events of emotional stress is presented in Section 4. Results are presented in Section 5 by evaluating each biomarker in terms of wearability, availability, and potential and future directions. Section 6 discusses the implications of the work, while Section 7 concludes the work and provides a guideline for future research.

## 2. Method

**Figure 1:**
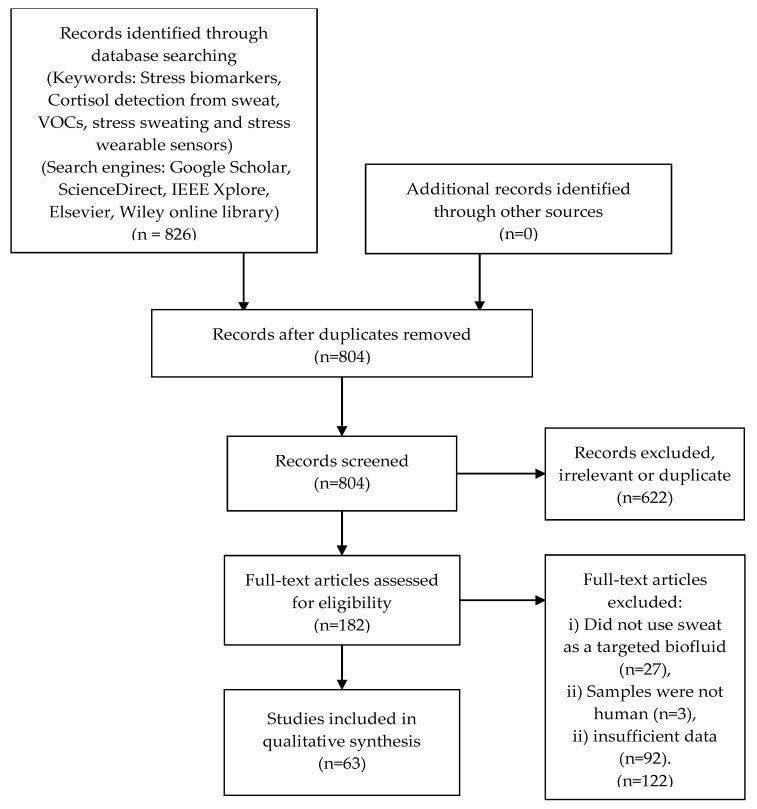
PRISMA process adopted and the results obtained in this review.

The design and methods used for this structured review comply with the preferred reporting items for systematic reviews and meta-analyses (PRISMA) guidelines [23]. Regarding eligibility criteria, we accepted all types of design and research outputs, and no restrictions were applied to samples. The followed PRISMA guidelines results are presented in Figure 1.

## 3. Sweat

### 3.1. Emotional Sweating Physiology

Perspiration’s main role is to maintain the core temperature of the human body at optimum levels, which is important for survival, as increasing the core temperature to over 40 °C causes serious health issues and can lead to death. In other words, the main objective of sweating is the downregulation of the body’s core temperature in high-temperature environments or under physiological stress. However, there are other major roles for sweating, including gustatory sweating and emotional sweating [24]. Regarding emotional sweating, this occurs as a physical reaction against emotive stimuli such as stress [25]. In an event of exposure to acute stress, the human body initiates several behavioral and physiological responses, known as the fight-or-flight response, which includes several connected activated mechanisms that enhance survival in events of danger and maintain homeostasis. The sympathetic nervous system reacts to acute stress by sending adrenaline and noradrenaline signals that cause multiple physiological changes, such as increases in heart rate, blood pressure, and breathing rate [26].

In a slightly slower timeframe, the hypothalamic–pituitary–adrenocortical (HPA) axis is activated, resulting in a production of the stress hormone cortisol as a part of increasing the circulation of glucocorticoids [26]. Emotional sweat is produced on the entire surface of the skin, but it is concentrated on the palms, soles, and underarms. All of these responses are relative, and as such the level of response is based on several factors, including the nature of the stressor and the stressed person [25]. Sweat from the palms and soles is usually caused by emotive stimuli, not by environmental temperature [27]. In comparison to thermal sweating, which can be affected by ambient temperature, emotional sweat does not change in response to the surrounding environment temperature. It increases dependently and decreases during mental repose and sleep [24]. On the other hand, similar to thermal sweating, sole and palm emotional sweating involves the eccrine glands [27]. However, there is a lack of information regarding the central pathway of the eccrine glands, although some evidence has shown that the cortex and amygdala are involved [28]. Interestingly, emotional sweating of the axillary area does not occur before pubescence, suggesting apocrine and apoeccrine glands play key roles in axillary emotional sweating, as they are inactive before this stage [29]. Apocrine glands are activated by adrenergic stimulation and strongly respond to emotion [30]. However, the function of the secretion in these glands is unclear yet, although there is evidence that apocrine odors have similar effects to pheromones [4].

### 3.2. Electrochemical Biomarkers from the Sweat

The human sympathetic nervous system reacts to stress through many physical and emotional reactions, which are collectively termed the fight-or-flight response. This response is activated from the sympathetic nervous system and adrenal medulla by several mediators, such as noradrenaline, leading to the production of cortisol from the adrenal cortex [31] and adrenaline [32]. However, there are different types of stressful situations, including the fight-or-flight response, acute stress, and chronic stress, causing the human body to react in many ways. In this regard, other hormones are produced in events of stress, such as corticotropin-releasing factor (CRF), adrenocorticotropic hormone (ACTH), and urocortin [33]. In addition, in response to stress, the levels of many hormones, such as insulin and growth hormone, are altered to adapt to the new circumstances [34].

**Figure 2:**
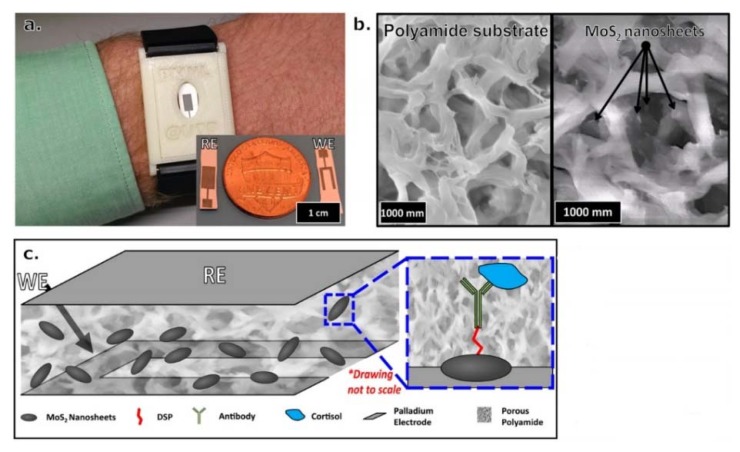
(**a**) Visualized wristband device prototype for monitoring cortisol in human sweat. (**b**) Scanning electron microscope (SEM) image of blank polyamide membrane on the left side, where MoS2 nanosheets were placed into porous polyamide membrane on the right side. (**c**) Stack of MoS2 nanosheets within a polyamide membrane sensing platform for cortisol detection. The blue box is a magnified picture of a nanosheet that presents the affinity assay for cortisol. Reproduced from Kinnamon et al. [38].

The salivary cortisol test has been identified as the most effective and promising noninvasive method to measure the cortisol level from biofluids [12] in concentrations ranging between 8.16 to 141.7 ng/m [35]. Most recently, sweat has started to be an attractive area of research for measuring cortisol [36]. In 2016, researchers developed a wearable device using nanosheets of zinc oxide (ZnO) to detect cortisol in sweat at concentrations of 1 to 200 ng/mL. The study used a thiol-based linker molecule to bind to the ZnO [37]. For low levels of cortisol volume detection, a portable cortisol sensor was developed using MoS2 sheets integrated into a nonporous flexible electrode system, as can be seen in Figure 2. The system succeeded in detecting volumes in the range of 1–5 μL. An affinity assay was designed, using MoS2 nanosheets operationalized with cortisol antibodies [38].

Most recently, CortiWatch, which is a wearable wristband with a watch shape, was developed for monitoring cortisol fluctuations within the physiological range (8–151 ng/mL) for 9 h. Although this device is a significant achievement in the field, it was designed to be disposed of after a low number of readings has been taken. The device has the potential to improve some medical applications, such as creating a circadian profile for a user and providing proof that self-monitoring of cortisol levels is possible [39]. Another recent study introduced an immunosensor that can detect cortisol and lactate using the label-free electrochemical chronoamperometric technique. This technique involves bioconjugation of cortisol and lactate antibodies with electro-reduced graphene oxide e-RGO, which is utilized as a synergetic platform for signal amplification. The prototype device can connect to smartphones via Bluetooth and can detect responses at concentrations as low as 0.1 ng/mL. In terms of selectivity, the device showed no cross-sensitivity between the two biomarkers or other components present in sweat [40]. Additionally, another cortisol detection immunosensor was introduced in a study using a miniaturized potentiostat (M-P) chip (LMP91000) to perform a three-electrode range cyclic voltammetry (CV) measurement. The system succeeded in detecting cortisol in the physiological range, with a sensitivity level of 1.24 μM of cortisol [41]. Additionally, a four-channel electrochemical impedance spectroscopy (EIS) analyzer module was designed to detect cortisol in sweat. This module utilizes flexible chemi-impedance sensors and was constructed with three gold electrodes for wearability. It was developed to detect cortisol in an ultra-low volume of sweat (1–3 μL) using an antibody-based technique, as well as to measure other physiological parameters, namely pH and skin temperature [42].

**Figure 3:**
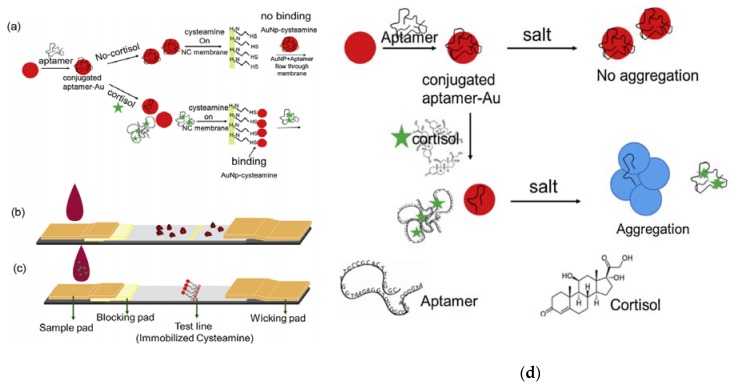
(**a**) Cortisol detection utilizing AuNP-aptamers: (**b**) negative control, for which there is no change in color in the absence of cortisol; (**c**) color change when cortisol is present; (**d**) process of cortisol effectiveness in releasing aptamers following salt aggregation. Reproduced from Dalirirad and Steckl [43].

In addition to antibody-based methods, an alternative technique has been developed to detect cortisol in sweat. This technique is a colorimetric detection method based on the conjugation of cortisol selective aptamers with the surfaces of gold nanoparticles (AuNPs). The aptamers react with cortisol molecules present in the sweat, provoking their desorption from the surface of the gold nanoparticles, resulting in reddish color in AuNPs, as can be seen in Figure 3. The changes in color are due to the introduction of salt in the colloidal solution, which causes aggregation of AuNPs. The sensor detects within the physiological range of cortisol present in sweat, using the visual range of detection (1 ng/mL). There were no interactions with other biomarkers in sweat. Moreover, the aptamers technique has advantages over antibody-based methods in terms of stability, costs, and user-friendliness [43]. In addition, e-nose also has been involved in cortisol detection. A study used e-nose with in association with a pattern recognition software tool to detect a low concentration of cortisol (5 μM–50 μM) in sweat [44]. In addition, Parlak et al. [45] presented a new patch-type multifunctional layered biosensor that was developed to detect cortisol in sweat. A molecularly imprinted polymer (MIP)-based artificial recognition membrane was developed to interpose between semiconductor polymer channels, typically the poly(ethylenedioxythiophene)–poly (styrenesulfonate) (PEDOT–PSS) channel layer, and the sweat reservoir to control the molecular transport (which is selective to cortisol) immediately from the skin to the organic electrochemical transistor (OECT) sensing channel. This molecularly selective OECT (MS-OECT) showed physical and chemical stability at body temperature, as well as the ability to counteract physical deformation. The device succeeded in detecting cortisol at low concentrations (0.1–1 μM) and no errors were reported concerning selectivity and specificity. Finally, Mugo and Alberkant [46] introduced a flexible nonenzymatic biometric molecularly imprinted electrochemical (MIP) sensor to detect cortisol in sweat. The sensor was fabricated using layer-by-layer (LbL) assembly and based on flexible poly(glycidylmethacryate-co ethylene glycol dimethacrylate)(poly (GMA-co-EGDMA)). The MIP was built to suit the human skin as a wearable device, as well as to be selective for cortisol detection in human sweat. The sample was collected from the forehead of one volunteer after exercise. The experiment was repeated for both a MIP sensor and a nonimprinted polymer (NIP), namely a cortisol-free labelled film that was polymerized similarly to the MIP but without the addition of cortisol. The selectivity of the sensor has been shown to be blind to other interfering sweat components. In terms of selectivity, the MIP sensor succeeded in detecting cortisol effectively in human sweat in the range of 10–66 ng/mL. However, the sensor has a limitation in terms of detecting cortisol at a lower range (2.0 ± 0.4 ng/mL). In comparison with the aptamers technique, the MIP technique is more economic and specific.

Regarding alternative biomarkers, a study also discovered that cortisol’s downstream metabolites (the 20α/β-DHCN) in the eccrine glands can be utilized as stress biomarkers in parallel with cortisol and cortisone [34]. These biomarkers have stability in terms of production in reaction to stress. The concentrations of cortisol and its metabolites were not altered by variables such as temperature and pH. The only concern is that concentrations were affected by the presence of other enzymes produced during a stress event [47]. However, the method was only used to detect these biomarkers in the laboratory, but it has the potential to target biomarkers in the future for wearable biosensor studies. Another aspect to be taken into consideration is the use of antistress hormones as biomarkers for stress as they are involved in the body’s reaction to stress [33]. Oxytocin has been identified as an antistress mediator [48]. Recently, a biosensor was developed to detect oxytocin using Zn^+^ ions from biofluid [49]. However, the biosensor was developed to detect Zn ions and Cu ions in biofluids, and was not designed for stress detection. This raises the question of whether it could be modified to detect stress. Further feasibility studies are needed to address the advantages and disadvantages of this sensor and to compare it with the current options available for detecting stress.

## 4. Volatile Organic Components (VOCs)

**Figure 4:**
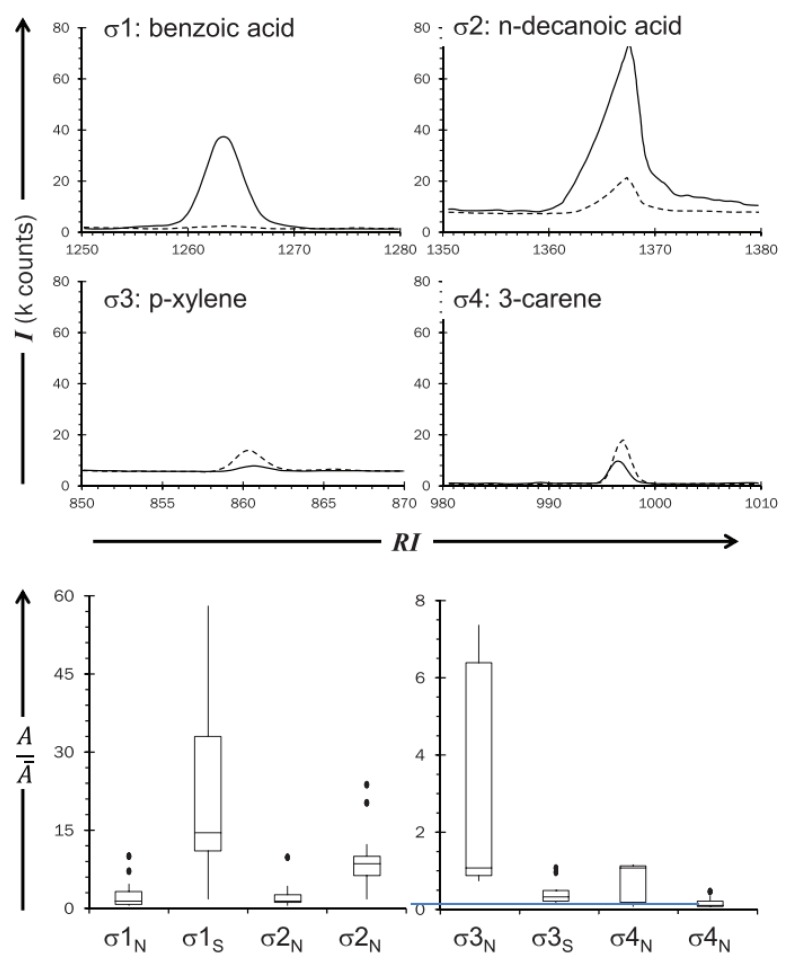
Overlaid extracted ion chromatograms from a sample from the four stress VOCs. Reproduced from Martin et al. [53].

The development of a noninvasive tool offering a significant level of selectivity and sensitivity with real-time operation is a challenging issue. For this reason, VOC sensing technology has been widely used in the medical field for several diseases that exhibit specific changes in the pattern of the VOCs of sweat [50]. Various gases are released from human bodies, including metabolic gases, while sweat VOCs and VOCs are produced by floral bacteria [51]. On the other hand, there is a lack of research on VOCs relating to human emotions, even though several studies have tested the role of sweat in human emotional interactions, such as fear sweat [52] and anger aggression [5]. These studies present the olfactory roles in emotional interactions, while the roles of chemical contents of emotional sweat had not been the focus of prior studies. Therefore, one study hypothesized that stress biomarkers are released from the skin in response to stress. The study used the trier social stress test (TSST) to measure stressors, a cortisol salivary concentration test as the gold standard for the study, and a survey as the result comparison tools. The gas analysis was performed by gas chromatography–mass spectrometry (GC/MS) system. The participants of this study were 30 females, as they are respond to TSST better than males. These subjects had a general anxiety tendency, which was evaluated using a physiological questionnaire. The subjects ranged between normal and high anxiety trait levels, reflecting the type of people who are likely to suffer from mental disorders as a result of stress. The study identified 6 stress biomarkers (1,2-ethanediol acetophenone, heptadecane, hexanedioic acid, dimethyl ester, benzyl alcohol, and benzothiazole) that were released from underarms of the samples [19]. Table 1 depicts the released amounts of the six stress VOCs that were identified as stress biomarkers in the skin. In the same vein, another study used a different methodology to identify stress VOCs. The paced auditory serial addition test (PASAT) was used as a stressor and sweat samples were collected from foreheads of 20 volunteers. The subjects were 10 males and 10 females between 19 and 26 years old. The samples were randomly separated into two sampling sessions. In the first session, subjects sat and listened to classical music. In the second session, subjects undertook the PASAT test. In addition, heart rate and blood pressure measurements were recorded. It was found that four stress biomarkers (benzoic acid, n-decanoic acid, a xylene isomer, and 3-carene) were present, as can be seen in Figure 4 [53]. Notably, the identified biomarkers from both studies were different. However, in terms of wearability, there are no commercial biosensors available to detect stress via VOCs, but a study did recommend a nanomaterial-based sensor array for future wearable biosensors for VOCs [54]. Unlike GC/MS, which identifies specific VOCs, this array relied on the collective pattern of VOCs.

## 5. Results

The results show a notable development in the field of electrochemical stress biosignals from sweat. Several methods have been utilized to detect cortisol, the main stress biomarker. In this regard, the antibody-based technique is the most common tool used to detect cortisol in sweat [37,39,40,41,55], while less commonly used techniques include the aptamer [43], e-nose [44,56], and MIP [45,46] techniques. In terms of sensitivity, all of the above-mentioned studies succeeded in detecting cortisol in its targeted range. However, different detection ranges were presented in the studies. The lowest levels of cortisol concentration detected were in the range 0.1 to 1.0 μM [45], while the only manufactured biosensor, CortiWatch, achieved a more modest level of detection, ranging from 1 to 150 ng/mL [39]. From the perspective of the placement of wearable sensor devices, it is an advantage that the eccrine glands are spread over the whole human body, as this offers a variety of placement options. In terms of selectivity, no reported errors were mentioned in cortisol biosensor studies.

Cortisol metabolites have the potential to be sensed as stress biomarkers in wearable devices. The current methods to detect them require sophisticated lab-based machines [47]. Further investigations are needed to create long-lasting sensors for wearable devices. Antistress hormones as stress biomarkers are also under-researched. However, a Zn^+^ ion biosensor has been developed to detect an antistress hormone called oxytocin in biological fluids for medical purposes [49]. From considering the literature, it is possible to recommend that more trial studies be conducted to detect the ranges and concentrations of biomarkers in sweat during a range of common stressful events, in order to further facilitate the capture of information needed in the design of biomarker sensors in future affective systems.

VOC technology is in the development stage. Two studies utilized different methodologies and found different stress biomarkers. The first study found changes in the concentrations of the biomarkers 2-hydroxy-1-phenylethanone, benzaldehyde, and 2-ethylhexan-1-ol in response to the stressor [53], while the second study found changes in the concentrations 1,2-ethanediol acetophenone, heptadecane, hexanedioic acid, dimethyl ester, benzyl alcohol, and benzothiazole [19]. However, in the first study, the biomarkers were found in the forehead samples, which might be produced from eccrine glands as cortisol metabolite VOCs, whereas in the second study the biomarkers were measured from apocrine glands (underarms). This difference might be the reason for the discrepancy between the experimental results obtained. Another possible reason is that the VOC biomarkers resulted from floral bacteria [19]. Further investigations are needed to understand the source of axillary VOCs, to also test the accuracy for both types of glands, and to test the performance of eccrine gland biomarkers at different places on the body.

Various signals have been identified as stress biomarkers. Table 2 summaries the results that were found in our research. Cortisol has been the most popular stress biomarker in sweat, with eight studies having targeted cortisol in the physiological range of sweat. Three techniques have been utilized to detect cortisol in experiments involving antibodies, aptamers, or e-nose technology. Antibody recognition methods including immunoassay and electrochemical immune sensing were utilized in five out of eight studies to detect cortisol [37,39,40,41,55]. These methods were effective in terms of specificity to cortisol molecules because of the nature of antibody–antigen immunochemistry [55]. CortiWatch [39], a cortisol wristband sensor, presents the antibody technique as an advanced step in this field. In terms of placement, the antibody-based methods detect cortisol from eccrine sweat, creating a promising future for cortisol detection technology, as eccrine glands are present on the whole surface of the body, which ensures the flexibility of manufacturing pervasive wearable devices. Alternatively, aptamer methods provide a visual, rapid detection method to detect cortisol in sweat [43]. The cortisol samples were, however, manufactured (i.e., no human body sample location was provided). Additionally, testing stress in real time has not been approved and finding suitable body placement locations for wearables have not been tested. Another cortisol detection method is e-nose, which “smells” the cortisol concentration in sweat vapors and uses additional pattern recognition tools to differentiate between stress events and quiet periods. Unlike the previous studies, sweat samples for this study were taken from the underarms of the samples, which means they were collected from apocrine glands. The sensitivity of the gas arrays increased directly with increasing cortisol concentration. However, a simplified wearable form of e-nose to detect cortisol concentration is not available. Samples were collected from apocrine glands (underarms), which could minimize the placements options, as apocrine glands are located in certain areas of the body, suggesting the potential for the development of wearable e-nose technology in “smart shirts” or armbands. Further studies are required to test e-nose technology for cortisol detection in eccrine glands, as succeeding in this would provide more fixable wearable options. 

The combined response to stress of cortisol, its metabolites, and cortisone raises the idea of using multiparameters rather than only using cortisol, as all these markers are present in sweat within a measurable range. By using GC/MS techniques, all the markers can be separated from each other, and also from other components of sweat, then variable concentrations and patterns can be measured in stressful events [45,47]. The samples in these studies were collected from eccrine glands, which indicates flexibility in terms of wearable device developments in the future. 

Antistress hormones are present in human biofluids during stress [33] but utilizing them as stress biomarkers is significantly under-researched. However, the antistress hormone oxytocin has several functions and indeed a biosensor has been developed to detect it, but not in stress detection events [48]. That might suggest utilizing oxytocin as a stress biomarker in future studies. Additionally, because its presence in biofluid has been already confirmed, confirming its presence in emotional situations should be further tested. 

In the first VOC study of its kind [53], benzoic acid, n-decanoic acid, a xylene isomer, and 3-carene were identified as stress biomarkers using the GC/MS lab technique. The sweat samples were collected from foreheads, which indicates that they are from the eccrine glands. In terms of wearability, this indicates the fixability of various places for monitoring. To detect VOCs in real time, e-nose and gas array sensors are commonly used, but unfortunately no device has been modified or developed to detect specific emotional VOCs. The second VOC study [19] found 1,2-ethanediol acetophenone heptadecane, hexanedioic acid, dimethyl ester, benzyl alcohol, and benzothiazole as stress biomarkers. While this work also used GC/MS tools, samples from the armpits (apocrine glands) were also collected. However, the source of VOCs collected from the axillary area is rather controversial, as they may be emitted by bacteria present in the collection area. Being limited to apocrine glands areas could minimize the options for developing wearable VOCs biosensors. Additionally, it is recommended to use human VOC sensors to detect the above-mentioned biomarkers using either e-nose or gas sensors.

## 6. Discussion

This paper has highlighted previous work, showing that the detection of sweat cortisol and VOCs emitted from the skin are effective methods for detecting stressful events, and have huge potential to supplement emotion detection systems in the future. Additionally, cortisol metabolites can be additional biomarkers to stress hormones that increase the efficiency of detecting emotional stress. Besides, antistress hormones can also potentially be used as stress biomarkers. Regarding cortisol detection using biochemical sensors, previous studies have shown three main methods, employing antibodies, aptamers, and MIPs. These methods have significant advantages over blood tests through classical laboratory techniques, as the latter requires a greater number of samples to be taken, consumes significantly more time, and needs trained staff to operate advanced tools [43]. In comparison between aptamers and antibody methods, aptamers are not rejected by the human immune system, as they are usually not considered foreign bodies, which makes them weakly immunogenic and toxic molecules, unlike antibodies that are highly immunogenic and toxic molecules [57]. Additionally, aptamers have more thermal stability than antibodies because of the nature of oligonucleotide-based aptamers, which can maintain their structure, while protein-based antibodies lose their structure at high temperatures. Therefore, aptamers can be used in various assay conditions [58]. Additionally, the production of aptamers is a cost-efficient approach compared to antibody production and allows for easier modification for different chemical reactions [59,60]. Lastly, for future stress biomarkers that may have weak immune responses, such that antibodies cannot be generated or produced, aptamers can be recommended, as they can detect ligands that antibodies cannot recognize [61]. However, in a comparison between MIPS and aptamers, MIPs seem to be more economic [62] and more specific in terms of target binding [63]. Generally, MIPs have advantages over all other recognition systems, as they have high selectivity, are inexpensive, have accurate mechanisms, and are environmentally stable, as can be seen in Table 3. Therefore, due to these advantages, MIPs have been widely used in several industries, including in chemical sensors and drugs [45].

However, the detection of cortisol directly from the sweat via e-nose technology is under-researched. In 2009, a study [44] showed a promising result, here e-nose detected stress situations by measuring the concentrations of cortisol and adrenaline in sweat; however, no further studies have been carried out on this. Alternatively, recent studies identified VOCs stress biomarkers emitted from the sweat or the skin during stress events [19,53]. However, the results of the two studies are controversial in many aspects. In the first study [53], samples were collected from eccrine glands (foreheads) and four stress biomarkers were found, while in the second study [19] no stress biomarkers were identified from the eccrine glands (palms), even though very similar methods (GC/MS) were used. This inconsistency raises the question of whether the eccrine glands are similar in different areas across the body. As some researchers have linked emotional sweating to the apocrine glands [19,64], it is also required to know if the eccrine glands produce emotional event VOCs. On the other hand, the source of stress VOCs identified by Tsukuda et al. [19] from the axillary area in the study is still unknown. The first possible source assumed was the apocrine gland, while the second possible source was floral bacteria. Addressing this issue may help find answers to the previous questions.

With respect to cortisol metabolites, they have been used as additional biomarkers for the stress hormones cortisol and cortisone for more accurate measurement. However, cortisol metabolites are only present 10 min after the production of cortisol in stressful events [47], which raises concerns regarding the effectiveness of utilizing them as biomarkers for acute stress, as this may require an immediate response. They may, however, be useful for less rapid stress situations or chronic stress conditions in mental health. Another challenge in this regard is that cortisol metabolites respond differently according to each individual, which suggests a need to develop techniques to deal with such individual differences [47]. 

With respect to antistress hormones, they are produced as a response to the production of stress hormones [33]. Oxytocin has been classified as an antistress hormone [48]. Although it has not yet been used as a stress indicator, its presence in biofluids has previously been detected [49]. More investigations are needed to check antistress hormone reliability as stress biomarkers, for example measuring the time between the production of stress hormones and antistress hormones. Additionally, although their presence in biofluid has been confirmed, their amounts in sweat must be confirmed in a measurable range.

Table 4 presents an analytical performance summary of the major biosensors reviewed in this work.

## 7. Conclusions

In this work, stress biomarkers were reviewed to present the current status of stress detection as an emotional event. In addition, potential biomarkers were also introduced for future studies. This paper has reviewed the electrochemical biomarkers of stress and highlights that cortisol is considered as a major stress biomarker because of its measurable presence in biofluids (sweat in this case), which makes it attractive to researchers. While most studies in this area have developed various methods of cortisol detection, this review also considered other possible stress biomarkers, including cortisol metabolites and antistress hormones, which are probably present in sweat as well. Another major focus of the work is volatile organic components (VOCs), which are have only just been considered in the most recent studies on stress detection. Studies has shown that there are a range of gasses emitted from different places on the skin, as demonstrated in various emotional stress tests. In several aspects, this field is still in the development stage. Firstly, the identified biomarkers from VOC studies are not yet coherent and different factors might be involved, such as stressors, placement, and types of glands. Secondly, all VOC experiments were measured in lab conditions; based on our knowledge, there are no currently wearable gas sensors available to sense human VOCs. However, some studies showed that e-nose or gas array sensors can smell environmental VOCs, as well as recognize human sweat cortisol concentrations by pattern recognition methods. It might be assumed that environmental VOCs biosensors can be modified to smell body odors. Also, pattern recognition for stress VOCs might be recommended for future studies. 

## Figures and Tables

**Table 1:** Released amount of volatile organic component (VOC) stress biomarkers. Reproduced from Tsukuda et al. [19]. AUC, area under the curve.

Compound	CAS No	*m/z*	Retention Time (min)	‘Under Stress Task’ vs. ‘Relax1’		‘Under Stress Task’ vs. ‘Relax2’	
				AUC Value	*p*-Value (Wilcoxon’s Sign Rank Test)	AUC Value	*p*-Value (Wilcoxon’s Sign Rank Test)
1,2-Ethanediol	107-21-1	33.1	25.6	0.82	<0.001	0.69	<0.001
Acetophenone	98-86-2	105	26.7	0.84	0.001 21	0.69	0.0019 23
Heptadecane	629-78-7	57.1	27.6	0.81	0.003 15	0.60	0.674 22
Hexanedionic acid, dimethyl ester	627-93-0	114.1	29.5	0.88	<0.001	0.74	0.0042
Benzyl alcohol	100-51-6	79.1	30.2	0.81	<0.001	0.75	<0.001
Benzothiazole	95-16-9	135	31.4	0.87	<0.001	0.66	0.153 65

**Table 2:** Summary of stress biomarkers from the sweat or skin, methods used to measure them, places flexibility, wearable device availability, and potential devices for future works. MIP, molecularly imprinted polymer; GC/MS, gas chromatography–mass spectrometry.

Biomarkers	Methods	Place	Wearable Available	Potential Device
Cortisol [37,39,40,41,43,44,45,55,56]	Antibodies, aptamers, e-nose, and the molecularly selective organic electrochemical transistor	Eccrine glands (antibodies, aptamers and MIPs) Apocrine (e-nose)	Wrist band + patch	e-nose + Flexible
Cortisol metabolites [34,47]	In labs only	Eccrine glands	No	Flexible
Stress antihormones [49]	Zn+ ions	Eccrine glands	No	Flexible
VOCs (study 1) benzoic acid, n-decanoic acid, a xylene isomer, and 3-carene [53]	Lab (GC/MS)	Eccrine glands (or skin) (forehead)	No	E-nose/gas array sensors
VOCs (study 2) 1,2-Ethanediol Acetophenone Heptadecane Hexanedioic acid, dimethyl ester Benzyl alcohol Benzothiazole [19]	Lab (GC/MS)	Underarms skin or apocrine glands	No	e-nose/gas array sensors

**Table 3:** Comparison between three cortisol detection techniques over several factors.

Factors/Techniques	Antibodies	Aptamers	MIP
Selectivity	High selectivity to cortisol—no errors have been reported	High selectivity to cortisol—no errors have been reported	High selectivity to cortisol—no errors have been reported
Sensitivity	In the physiological range	In the physiological range	The highest sensitivity (0.1 ng/mL)
Thermal stability	The lowest	High stability	The highest
Immune response	Can be rejected by the immune system	Cannot be rejected	Cannot be rejected
Cost	Expensive	Less expensive	Cheapest

**Table 4:** Analytical performance summary of the major biosensors reviewed.

Reference	Stress Biomarker	Technique	Concentration	Volume	Within the Physiological Range of 8.16 to 141.7 ng/m? (Yes/No)
[37]	Cortisol	Cortisol antibodies	1 ng/mL to 200 ng/mL	N/A	Yes
[38]	Cortisol	Cortisol antibodies	N/A	1–5 μL	Yes
[39]	Cortisol	Cortisol antibodies	1 ng/mL to 150 ng/mL	N/A	Yes
[40]	Cortisol	Cortisol antibodies	0.1 ng/mL	N/A	Yes
[41]	Cortisol	Cortisol antibodies	1.24 μM	N/A	Yes
[42]	Cortisol	Cortisol antibodies	N/A	1–3 μL	Yes
[43]	Cortisol	Cortisol aptamers	1 ng/mL	N/A	Yes
[44]	Cortisol	E-nose	5 mL–50 mL	N/A	Yes
[45]	Cortisol	MIPs	0.1 μM–1 μM	N/A	Yes
[46]	Cortisol	MIPs	10 ng/mL–66 ng/mL	N/A	Yes
[19,53]	Stress VOCs	GC/MS	N/A	N/A	N/A
